# The biopsychosocial impact of hypermobility spectrum disorders in adults: a scoping review

**DOI:** 10.1007/s00296-023-05298-2

**Published:** 2023-03-10

**Authors:** Natalie L. Clark, Melissa Johnson, Amar Rangan, Lucksy Kottam, Katherine Swainston

**Affiliations:** 1 South Tees Hospitals NHS Foundation Trust, Middlesbrough, UK; 2 The Mary Kinross Trust and RCS Chair, Department of Health Sciences and Hull York Medical School, University of York, York, UK; 3 School of Psychology, Faculty of Medical Sciences, Newcastle University, Newcastle Upon Tyne, UK

**Keywords:** Benign hypermobility syndrome, Hypermobility, Joint, Ehlers-Danlos syndrome, Models, Biopsychosocial, Quality of life

## Abstract

Joint hypermobility affects approximately 30% of the United Kingdom (UK) population, characterised by the ability to move joints beyond the physiological limits. Associated conditions include Ehlers-Danlos syndrome and hypermobility spectrum disorders, affecting individuals across physical, psychological and social levels detrimentally impacting their health and wellbeing. The scoping review aims to describe the known biopsychosocial impact of joint hypermobility conditions in adults over the last decade. Additional objectives include to (1) identify the types of studies that address these factors, (2) to understand how the impact of the condition is measured and managed and (3) what healthcare professionals (HCPs) are involved. The scoping review was conducted using the five-stage framework by Arksey and O’Malley. The search strategy related to two main keywords, “hypermobility” and, “biopsychosocial” across a number of electronic databases. A pilot search was conducted to determine the suitability of the databases and terms. Following the search, the data was extracted and charted, summarised and narratively reported. 32 studies met the inclusion criteria. The majority were conducted in either the UK or United States of America and case–control in design. The biopsychosocial impact was wide-ranging including, but not limited to, musculoskeletal system and dermatology, gastroenterology, mood and anxiety disorders, education and employments. This review is the first of its kind to summarise all reported symptoms and impact of joint hypermobility conditions in adults, highlighting a clear need to promote a multidisciplinary and holistic approach in raising awareness of these conditions and improving their management.

## Introduction

Joint hypermobility is primarily characterised by the ability to move a joint beyond what is considered to be within the normal range of motion [1], reported to affect around 30% of the United Kingdom (UK) population [2]. The two most common conditions linked to joint hypermobility, often viewed as indistinguishable from each other, are Ehlers-Danlos syndrome [(EDS), specifically hypermobile EDS (hEDS)], an inherited connective tissue disorder [1, 3], and hypermobility spectrum disorders (HSD) [3]. The prevalence of EDS and HSD varies, one study collecting data in Wales (UK) reported a prevalence of 0.2% [4], whilst another estimated it to be 3% in the UK general population [5].

Both EDS and HSD affect individuals across physical (biological), psychological and social levels [6] with the predominant physical manifestations being joint dislocations and musculoskeletal (MSK) pain [7, 8]. Fatigue, headaches, dizziness, and gastrointestinal symptoms are also frequently reported symptoms [7, 8]. Individuals have also been found to be more likely diagnosed with a psychiatric disorder such as depression or anxiety [9] and experience depressive symptoms and feelings of angst [8]. These varied physical and psychological manifestations consequently impact the social life of an individual with EDS/HSD contributing to social isolation, financial problems, and strained relationships [8].

This is just a small insight into some of the presenting complaints and the adverse consequence on an individual’s health, quality of life (QoL) and wellbeing. Due to the wide-ranging presentations, there is understandably a lack of awareness and understanding amongst healthcare professionals (HCPs). Diagnosing either EDS or HSD is quite challenging due to the varying symptoms across multiple levels. Frequently, individuals face a long diagnostic journey, some waiting as long as 10 years [8], whilst others are often misdiagnosed, both leading to a deterioration in their health [1, 7]. Considering this, the reported prevalence is believed to be much higher than recorded in current literature, due to misdiagnosis or underreporting [4]. Training HCPs across specialities (e.g., gastroenterologists) to recognise EDS and HSD presentations could reduce the adverse health and wellbeing consequences caused by a delayed diagnosis. For example, skin hyperextensibility is another known symptom of EDS and HSD, it has therefore been suggested that dermatologists could be trained to recognise, diagnose and/or refer for appropriate management [10].

The aim of this scoping review is to map the biopsychosocial impact reported in adults with EDS and/or HSD. The biopsychosocial approach [11] acknowledges the whole impact of the condition on an individual beyond just the physical manifestations, including the symptoms of a psychological and social nature and how each manifestation interlinks with one another. How the symptoms and QoL is measured and managed, as well as HCP involvement, will also be mapped. In doing so, this will increase understanding of the condition, knowing which HCPs should be trained to recognise the condition to aid early diagnosis and develop appropriate interventions to aid its effective management.

## Methods

A protocol for the review has been written and submitted prior to commencing the review [12]. The review has been conducted and reported using the five-stage framework by Arksey and O’Malley [13]: (1) identifying the research question; (2) identifying relevant studies; (3) study selection; (4) charting the data; (5) collating, summarising and reporting the results. The optional sixth step for consulting with stakeholders to inform or validate the findings was not utilised for this scoping review. The search methodology also followed the recommendations of Gasparyan et al. [14] for a biomedical review.

### Identifying the research question

The primary research question was developed following the PCC (population, concept, context) framework as recommended by the Joanna Briggs Institute [15]: “What evidence exists on the biopsychosocial impact of EDS and HSD in the adult population?”.

The objectives of the scoping review were to:

1. Map the known evidence of the biopsychosocial impact of EDS/HSD in the adult population.
2. Identify and report the types of studies used to identify the biopsychosocial impact.
3. Identify and describe how the biopsychosocial impact is measured and managed.
4. Identify and describe the HCP involvement.

### Identifying relevant studies

The search terms used related to two main keywords, “hypermobility” and, “biopsychosocial” in combination with the Boolean terms, “AND” and, “OR”. Eight electronic databases were searched: MEDLINE, EMBASE, AMED, CINAHL, PsycINFO, Cochrane Library, PubMed, PEDro. A secondary search of clinical trials and study protocols was conducted in clinicaltrials.gov, EU clinical trials register and the ISRCTN registry. A final hand search of reference lists of the accepted articles was later conducted. The search strategies across the databases can be viewed in Table 1.

**Table 1:** Electronic database search

Date of search	Electronic database	Keywords	Limits	No. studies retrieved	No. studies for review
30th May 2022	Ovid MEDLINE (Embase)	“hypermobility” OR “Benign Joint hypermobility” OR “Ehlers Danlos Syndrome” OR “hypermobile” OR “Hypermobility Spectrum Disorder” OR “joint hypermobility” OR “Joint Hypermobility Syndrome” OR “Generalised Joint Hypermobility” AND “lived experience” OR “psychological” OR “psychosocial” OR “psychology” OR “social” OR “symptoms” OR “biopsychosocial” OR “quality of life”	Publication year: 2012–2022 English language	704	129
30th May 2022	Ovid Embase	“hypermobility” OR “Benign Joint hypermobility” OR “Ehlers Danlos Syndrome” OR “hypermobile” OR “Hypermobility Spectrum Disorder” OR “joint hypermobility” OR “Joint Hypermobility Syndrome” OR “Generalised Joint Hypermobility” AND “lived experience” OR “psychological” OR “psychosocial” OR “psychology” OR “social” OR “symptoms” OR “biopsychosocial” OR “quality of life”	Exclude MEDLINE Publication year: 2012–2022 English language	225	22
30th May 2022	AMED	“hypermobility” OR “Benign Joint hypermobility” OR “Ehlers Danlos Syndrome” OR “hypermobile” OR “Hypermobility Spectrum Disorder” OR “joint hypermobility” OR “Joint Hypermobility Syndrome” OR “Generalised Joint Hypermobility” AND “lived experience” OR “psychological” OR “psychosocial” OR “psychology” OR “social” OR “symptoms” OR “biopsychosocial” OR “quality of life”	Publication year: 2012–2022	24	8
30th May 2022	CINAHL	“hypermobility” OR “Benign Joint hypermobility” OR “Ehlers Danlos Syndrome” OR “hypermobile” OR “Hypermobility Spectrum Disorder” OR “joint hypermobility” OR “Joint Hypermobility Syndrome” OR “Generalised Joint Hypermobility” AND “lived experience” OR “psychological” OR “psychosocial” OR “psychology” OR “social” OR “symptoms” OR “biopsychosocial” OR “quality of life”	Publication year: 2012–2022 English language	256	64
30th May 2022	APA PsychInfo	“hypermobility” OR “Benign Joint hypermobility” OR “Ehlers Danlos Syndrome” OR “hypermobile” OR “Hypermobility Spectrum Disorder” OR “joint hypermobility” OR “Joint Hypermobility Syndrome” OR “Generalised Joint Hypermobility”	Publication year: 2012–2022 English language	131	30
30th May 2022	Cochrane Library	“joint hypermobility” OR “Ehlers Danlos Syndrome” AND “psychology” OR “lived experiences”	Publication year: 2012–2022	46	5
30th May 2022	PubMed	“hypermobility” OR “Benign Joint hypermobility” OR “Ehlers Danlos Syndrome” OR “hypermobile” OR “Hypermobility Spectrum Disorder” OR “joint hypermobility” OR “Joint Hypermobility Syndrome” OR “Generalised Joint Hypermobility” AND “lived experience” OR “psychological” OR “psychosocial” OR “psychology” OR “social” OR “symptoms” OR “biopsychosocial” OR “quality of life”	Publication year: 2012–2022 English language	53	34
30th May 2022	PEDro	“joint hypermobility”	Since 2012	12	0
30th Jul 2022	Clinicaltrials.gov	“hypermobility”	Adult (18–64) Older adult (65 +) Study start: 2012–2022	44	1
30th Jul 2022	EU Clinical Trials Register	“hypermobility”	Adult Date range: 2012–2022	2	0
30th Jul 2022	ISRCTN Register	“hypermobility”	Overall trial start date: 2012–2022	4	0
30th Jul 2022	Reference lists of accepted articles	–	–	18	18

To determine the suitability of the search terms and electronic databases, a pilot search was conducted by two of the authors (NC and MJ). The pilot search was supported by an academic librarian. Adjustments to the search terms were made as appropriate, including removing and adding new terms.

### Study selection

#### Eligibility criteria

The inclusion criteria for the review were:

- Adult sample (over 18 years old) with a clinical diagnosis (i.e. by a clinician or using validated tools) of a joint hypermobility condition (e.g. EDS or HSD).
- Study designs investigating the physical (biological), psychological and/or social impact (e.g. cross-sectional, qualitative, case studies).
- Recent literature published between 2012 and 2022, to enhance relevance of the findings to current clinical practice.

Articles were excluded from the review if:

- Inaccessible full-text articles.
- Full-texts unavailable in the English language.
- Systematic or literature reviews not meeting the eligibility criteria.

#### Selection process

**Fig. 1:**
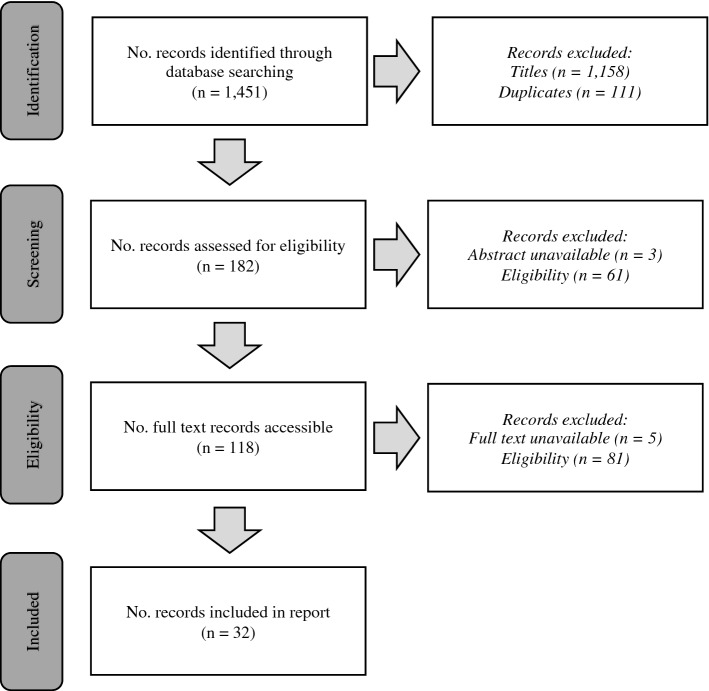
Flow diagram of study selection process

The search identified 1451 articles, of which 293 were exported following a title review. Duplicate articles were removed, leaving 182 articles for abstract review. Articles were excluded if they did not meet the inclusion criteria or abstracts were inaccessible once all attempts to retrieve abstracts were unsuccessful. Following a review of the abstracts, 118 of the articles were eligible for a full-text review, any exclusions had the reasons documented, as reviewed by two of the authors (NC and MJ). In addition, reference lists of the 32 accepted full-text articles were screened for articles that might have been missed during the database search, this resulted in 18 articles to screen and none eligible. Any uncertainties or disagreements were resolved through discussion with a third author (KS). Figure 1 represents the study selection process using a PRISMA-ScR flow diagram [16].

### Charting the data

The data extraction tool was developed with headings that would accurately address the primary research question and objectives. A pilot data extraction was conducted by two of the authors (NC and MJ) during the pilot phase. The process was discussed and amendments to the tool were made as necessary to refine data collection. Queries arising during the data extraction process were discussed with the relevant senior authors (KS, LK and AR), appropriate to their area of expertise.

Data was extracted according to: (1) authors, year and country; (2) study design; (3) participant characteristics (sample size, gender, mean age and standard deviation, EDS/HSD diagnosis, recruitment date); (4) physical (biological) symptoms or conditions; (5) psychological symptoms or conditions; (6) social symptoms or conditions; (7) findings; (8) HCP involvement; (9) measurements; (10) management or treatment.

### Collating, summarising and reporting the results

Data obtained within the extraction tool was collated, summarised and reported to narrate the biopsychosocial impact, symptoms and associated conditions adults with EDS/HSD. This has been presented in tabular format to supplement the narrative summary. The data was also collated, summarised and reported to acknowledge the HCP involvement, measurements and management/treatment. Verification of the data was conducted by three of the authors (NC, MJ and KS).

## Results

### Characteristics of included studies

Table 2 describes the characteristics of the 32 included studies. The majority of studies were conducted in either the UK or United States of America (USA) (*n* = 6, 19%), followed by France (*n* = 4, 13%) and were mostly case–control (*n* = 10, 31%) or cross-sectional (*n* = 8, 25%) in design. The sample size ranged from a total of 1 to 2404, with the majority (*n* = 31, 97%) having a predominantly female sample and a mean age range of 20–68.5 years (*SD* = 5.71–13.9).

**Table 2:** Study characteristics

Author (year), Country	Study design	Participant characteristics				
		*N*	Female (%)	Mean age (SD)	HSD diagnosis (%)	Recruitment dates
Palomo-Toucedo, et al. (2020), Spain [6]	Qualitative	26	80.8	41.3	EDS	Apr 2018–Sep 2019
Hershenfeld, et al. (2016), Canada [9]	Retrospective cohort	106	84.9	35.2 (13.9)	cEDS (16) hEDS (67.9) c/hEDS (16)	2007–2013
Alomari, et al. (2020), USA [16]	Retrospective cohort	218	90.8	32.3	hEDS/EDS-HT	Jan 2007–Dec 2017
Baeza-Velasco, et al. (2018), France [17]	Cross-sectional (observational)	80	90	37.1	hEDS	NS
Murray, et al. (2013), USA [18]	Cross-sectional	466	89.9	NS	EDS-HT	Sep 2009–Apr 2010
Halverson, et al. (2021), USA [19]	Qualitative	22	77	38	hEDS (86)	Jan–Aug 2020
Maya, et al. (2021), USA [20]	Retrospective cohort	144	93.8	31	hEDS (41.7) HSD (58.3)	Jan 2017–Jan 2020
Wasim, et al. (2019), Canada [21]	Retrospective cohort	391	85.2	36.1 (14.4)	HSD (79.8) cEDS (11.3) clEDS (7.9) Other EDS (a, v, k) (1)	Jan 2013–Jun 2016
Saetre & Eik (2019), Norway [22]	Qualitative	7	85.7	34.6	JHS EDS-HT	NS
Terry, et al. (2015), UK [23]	Qualitative	25	88	38.2	JHS EDS-HT	Jan 2013–Feb 2013
Mullick, et al. (2013), India [24]	Case report(s)	84	46.4	30 (5.71)	BJHS	May 2010–May 2011
Martinez, et al. (2021), USA [25]	Case–control (cross-sectional)	148 (23 unaffected, 27 with HSD, 98 with hEDS)	7.4 (unaffected) 75 (HSD/hEDS)	50 (unaffected) 37.8 (HSD) 40.9 (hEDS)	HSD hEDS	NS
Folci & Capsoni (2016), Italy [26]	Case report(s)	1	100	20	JHS	Jul 2014
Baeza-Velasco, et al. (2016), France [27]	Case report(s)	2	100	22	JHS EDS-HT	NS
Lee & Strand (2017), Sweden [28]	Case report(s)	1	100	23	EDS	NS
Berglund, et al. (2015), Sweden [29]	Cross-sectional	250	89	46.2	EDS not defined (38) hEDS (30) cEDS (18) aEDS (1) vEDS (4) mEDS (9)	2008
Clark, et al. (2014), UK [30]	Case–control (prospective cohort)	203 (90 JHS, 113 healthy volunteers)	92.2 (JHS) 75.6 (healthy volunteers)	33.96 (9.94—JHS) 35.73 (13.24—healthy volunteers)	JHS	May 2009–Aug 2009
Inayet, et al. (2018), UK [31]	Case–control	180 (45 MS, 45 with hEDS, 90 controls)	73 (MS) 86 (hEDS) 80 (controls)	28 (MS) 24 (hEDS)	MS hEDS	NS
Albayrak, et al. (2015), Turkey [32]	Case–control	229 (115 BJHS, 114 controls)	88.7 (BJHS) 86.8 (controls)	30.17 (7.47—BJHS) 31.81 (6.86—controls)	BJHS	April 2013–Dec 2013
Gaisl, et al. (2017), Switzerland [33]	Case–control	200 (100 EDS, 100 control)	82	39.9 (12.8—EDS) 39.8 (12.4—control)	EDS	NS
Zeitoun, et al. (2013), France [34]	Cross-sectional	134	91	35 (14.7)	cEDS (8.2) hEDS (80.6) vEDS (2.2) Other (9)	Sep 2011–Jul 2012
Fikree, et al. (2017), UK [35]	Case–control (observational)	289 (30 JHS, 259 controls)	93 (JHS) 45.2 (controls)	29.5 (JHS)	JHS	Jan 2010–Dec 2011
Mastoroudes, et al. (2012), UK [36]	Case–control (cross-sectional)	120 (60 BJHS, 60 controls)	100	39.4	BJHS	Oct 2010–Aug 2011
Mastoroudes, et al. (2013), UK [37]	Case–control	120 (60 BJHS, 60 controls)	100	39.4	BJHS	Oct 2010–Aug 2011
Puledda, et al. (2015), Italy [38]	Case–control	99 (33 JHS/EDS-HT, 66 migraine controls)	87.9	32.11 (11.63—JHS/EDS-HT) 32.39 (11.7—migraine controls)	JHS EDS-HT	NS
Bulbena-Cabre, et al. (2018), Spain [39]	Cross-sectional	108	55	68.5 (men) 67.4 (women)	JHS	NS
Baeza-Velasco, et al. (2022), France [40]	Cross-sectional	35	100	39.7 (13.9)	hEDS	Mar 2015
Liaghat, et al. (2022), Denmark [41]	Cross-sectional	100	79	37.8	HSD	Apr 2019–Dec 2020
Berglund & Björck (2012), Sweden [42]	Case–control	769 (250 EDS, 519 controls)	89.2 (EDS) 49.9 (controls)	46.1 (EDS)	EDS EDS-HT EDS-MT EDS-UT EDS-VT EDS-AT	2008
De Baets, et al. (2021), Belgium [43]	Qualitative	9	89	40.5	hEDS (100)	NS
Nee, et al. (2019), USA [44]	Cross-sectional (observational)	2404 (1804 EDS, 600 with MS)	93.7 (EDS) 61 (MS)	40.9 (EDS) 44.5 (MS)	EDS MS	Oct 2014–Jan 2015
Bisaralli, et al. (2017), India [45]	Case report(s)	3	100	26.7	BJHS	NS

*aEDS* artrocalasia Ehlers-Danlos syndrome; *BJHS* benign joint hypermobility syndrome; *cEDS* classical Ehlers-Danlos syndrome; *clEDS* classical-like Ehlers-Danlos syndrome; *c/hEDS* classical/hypermobility Ehlers-Danlos syndrome; *EDS* Ehlers-Danlos syndrome; *EDS-AT* Ehlers-Danlos syndrome arthrochalasia-type; *EDS-HT* Ehlers-Danlos syndrome hypermobility-type; *EDS-MT* Ehlers-Danlos syndrome mixed-type; *EDS-UT* Ehlers-Danlos syndrome unknown-type; *EDS-VT* Ehlers-Danlos syndrome vascular-type; *hEDS* hypermobility Ehlers-Danlos syndrome; *HSD* hypermobility spectrum disorder; *JHS* joint hypermobility syndrome; *MS* Marfans syndrome; *mEDS* mixed Ehlers-Danlos syndrome; *NS* not stated; *SD* standard deviation; *UK* United Kingdom; *USA* United States of America; *vEDS* vascular Ehlers-Danlos syndrome

All studies included samples with a clinical diagnosis of either EDS (inclusive of sub-types) or HSD [e.g., joint hypermobility syndrome (JHS)] as diagnosed by an appropriate clinician or validated tool. Diagnosis was mostly done by rheumatologists [17], geneticists [17], national experts in EDS [18] or primary care practitioners [19], with referrals to mental health professionals not uncommon though viewed as dismissive by individuals [20]. The Beighton Scoring System (*n* = 10, 31%) and Brighton Diagnostic criteria (*n* = 9, 28%) were most frequently used as the validated tool to support a diagnosis of joint hypermobility whilst a more recently revised diagnostic criteria for EDS was less frequently utilised (*n* = 3, 9%) [17, 21, 22].

### Biopsychosocial impact

**Fig. 2:**
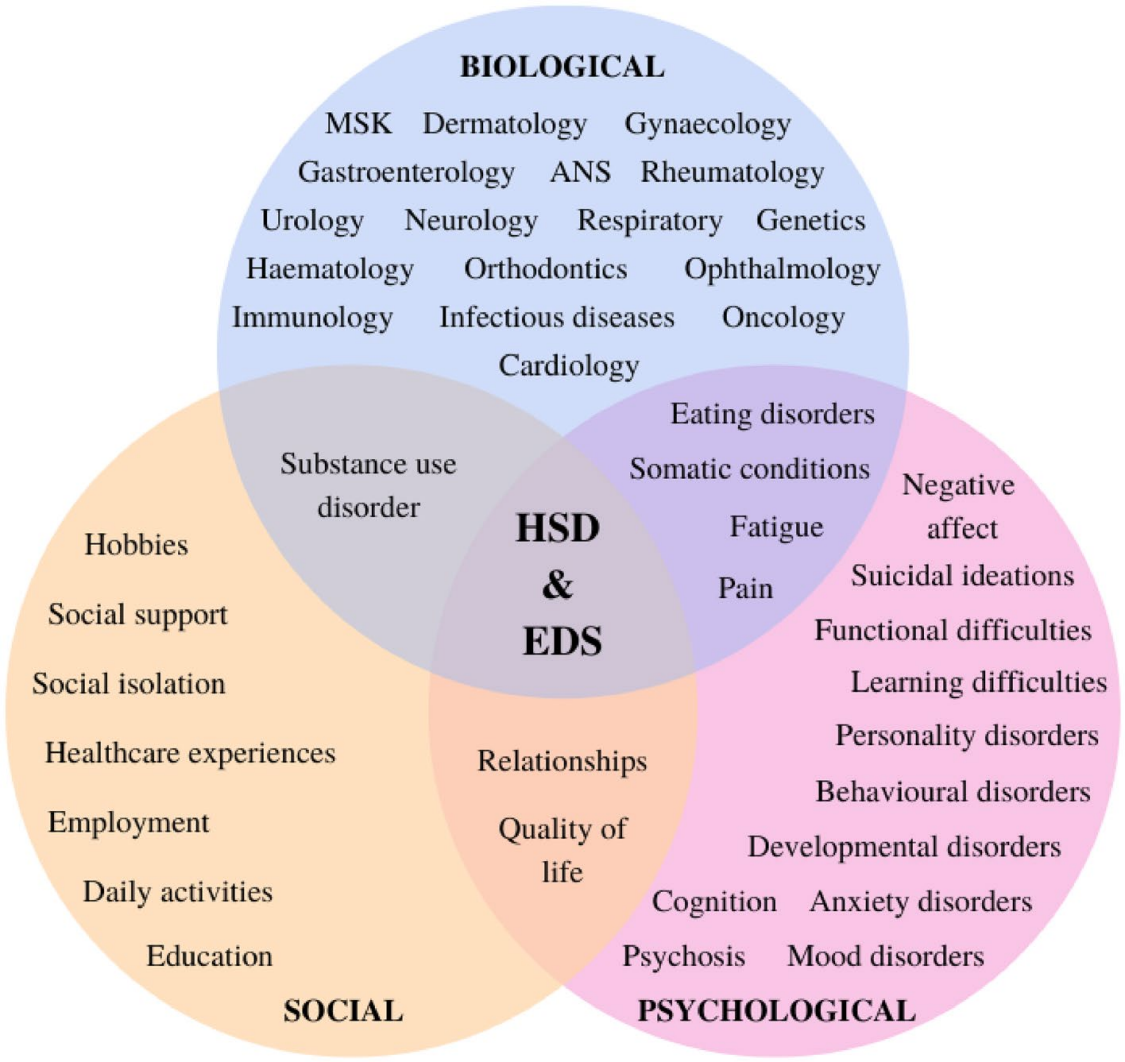
Venn diagram of the biopsychosocial impact of HSD/EDS

In this review, 31 (97%) of the included studies referred to physical manifestations whilst 29 (91%) studies referred to psychological manifestations. These manifestations were found to be either directly related to the EDS/HSD e.g., dysautonomia, or otherwise highly prevalent and clinically significant within this population e.g., anxiety and depression. Weak associations and less frequently reported symptoms and conditions have also been documented within this review for completeness. The social impact is referred to throughout the included studies (e.g., impact on QoL), more so in the qualitative studies where individuals had the opportunity to detail their lived experiences and diagnostic odyssey. The symptoms and conditions have been categorised by speciality sub-headings with Tables 3 and 4, and Fig. 2 providing more details.

**Table 3:** Biopsychosocial impact and study findings

Author (year)	Physical	Psychological	Social	Findings
Palomo-Toucedo, et al. (2020) [6]	Miscellaneous: pain, physical fatigue, exhaustion	Negative affect: exhaustion, misunderstood Miscellaneous: pain, fatigue	Employment: professional life Hobbies/daily activities: physical activity limited Lifestyle: restriction on daily routines, atmospheric changes Social relationships: social isolation behaviours, misunderstanding, social image, short term social plans, social relationships, sexual sphere, support groups/sharing, emotional support;	EDS affects daily life, in a physical level and a psychological and social sense. Social support is important. Patients need to be aware of the changes in their social life because of the disease so that they can have a better QoL. Progression of symptoms reduce employment opportunities, causing economic restrictions and diminish their self-care. HCPs need to know this information to provide better care and improve their QoL
Hershenfeld, et al. (2016) [9]	Cardiology Dermatology: skin hyperextensibility, abnormal scarring, striae Gastroenterology: abdominal pain, functional bowel disorders Haematology MSK/Orthopaedics: joint pain, inflammation, hypermobility, dislocations and subluxation, muscle pain Neurology: neuropathic pain, headaches, migraines Miscellaneous: pain symptoms, fatigue	Anxiety disorders (23.6%): PTSD (4.7%) Behavioural disorders: ADHD (6.6%), conduct disorder (1.9%) Developmental disorder: Asperger disorder (0.9%) Eating disorders: AN (1.9%) Learning difficulties (1.9%) Mood disorder: depression (42.5%), bipolar disorder (1.9%); Personality disorder: BPD (3.8%), schizotypal disorder (0.9%) Psychosis: schizoaffective disorder (0.9%) Miscellaneous: fatigue	NS	High frequency of psychiatric disorders (e.g., depression and anxiety disorders). Pain symptoms are significantly associated with having a psychiatric disorder. Need to screen for mental health disorders in EDS patients, especially with pain symptoms. Need to support the recognition of psychiatric disorders within EDS. Many are stigmatised and dismissed without further investigation, contributing to delays in a diagnosis of EDS. Awareness will better address, manage and improve QoL
Alomari, et al. (2020) [16]	ANS/Cardiology: PoTS (39.9%) Gastroenterology: *GI dysmotility:* oesophageal dysmotility (23.8%), gastroparesis (42.8%), small bowel/colon altered transit time (11.9%), global dysmotility (9.5%) *GI manifestations:* abdominal pain (49.8%), nausea (49.5%), constipation (45.4%), diarrhoea (37.6%), IBS (28%), IBS subtypes (D—4.1%, C—2.3%, M—2.8%, U—12.4%) bloating/belching (27.1%), vomiting (26.1%), dysphagia (14.2%), faecal incontinence (6%), heartburn (35.8%), pelvic floor dysfunction (33.9%), faecal urgency (3.7%), rectal prolapse (1.8%), GORD (37.6%), oropharyngeal dysphagia (1.4%) Rheumatology: fibromyalgia (35.8%)	Anxiety disorder: GAD (29.4%) Behavioural disorder: ADHD (11%) Mood disorder: depression (33%), bipolar disorder (6%)	NS	GI dysmotility in hEDS is not uncommon and underdiagnosed. PoTS was an independent predictive factor for GI dysmotility in hEDS. Need to improve outcomes and quality of care
Baeza-Velasco, et al. (2018) [17]	Miscellaneous: mild/moderate pain (32.5%), severe/very severe pain (66.25%), mild/moderate fatigue (31.25%), severe/very severe fatigue (68.75%)	Anxiety disorder: anxiety Cognitive: pain catastrophising Mood disorder: high depressive symptomatology (18.75%) Negative affect: fear of pain and movement Miscellaneous: somatosensory amplification	Social relationships: social functioning, social support	Important to consider the psychosocial factors–this will improve adjustment to this chronic condition and provide support to those affected via a biopsychosocial approach
Murray, et al. (2013) [18]	Cardiology (96%): tachycardia, palpitations Dermatology (95%): skin hyperextensibility, problems with scarring, smooth/velvety skin, easy bruising Gastroenterology (96%): rectal prolapse, IBS, GERD, diarrhoea, nausea, constipation Gynaecology/Urology (67%): uterine prolapse, bladder prolapse, infertility, endometriosis, vulvodynia MSK/Orthopaedics (99%): locked jaw, scoliosis, flat feet, arthritis, TMJD, joint dislocations, subluxations and pain, limb pain, hypermobility Neurology (88%): restless leg syndrome, hypersomnia, nerve compression, other headache, tension headache, migraine headache, dizziness Rheumatology: fibromyalgia Miscellaneous: chronic/constant pain (67%), tiring/exhausting pain (93%), aching (80%), chronic fatigue (92%), insomnia	Anxiety disorder: anxiety (73%) Mood disorder: depression (69%) QoL Miscellaneous: insomnia, chronic fatigue, dizziness, chronic/constant pain (67%), tiring/exhausting pain (93%), aching (80%), chronic fatigue (92%)	Education: unable to enrol full time Employment: change roles, less responsibility QoL	Documenting of the broad array of symptoms, clinical diagnoses and effects on QoL has important implications for HCPs. Supports the need for better recognition, understanding, and management as a multisystemic, disabling condition and a multidisciplinary approach to management is necessary
Halverson, et al. (2021) [19]	ANS/Cardiology: dysautonomia, PoTS (63.6%) Dermatology: rashes Endocrinology: Cushing syndrome (4.5%) Gastroenterology: vomiting, CVS (13.6%), gastroparesis (13.6%), ulcerative colitis (9.1%), IBS (4.5%) Gynaecology: endometriosis (9.1%), pelvic floor dysfunction (22.7%), PCOS Immunology: MCAS (40.9%) Infectious diseases: Epstein Barr (13.6%), Lyme disease (9.1%), meningitis (9.1%) MSK/Orthopaedics: hyperextending (arms and legs), extraordinary flexibility, chronic knee pain, stiffness, cervical instability (31.8%), carpal tunnel syndrome, scoliosis (13.6%) Neurology: recurrent headaches, neurosyncope, cognitive fogs, fainting, headaches, meningitis (9.1%), MS (22.7%), intercranial hypertensions (4.5%) Oncology: paraneoplastic syndrome (4.5%) Ophthalmology: glaucoma (4.5%) Respiratory: respiratory dystonia, stridor Rheumatology: fibromyalgia (40.9%), lupus (13.6%), Raynaud’s disease (9.1%) Miscellaneous: pain, fatigue, unbearable pain, chronic pain, iatrogenic physical harms, CRPS (9.1%), chronic fatigue (27.3%), tethered cord (9.1%), cerebellar tonsillar ectopia (4.5%), cytomegalovirus (4.5%), failed neck syndrome (4.5%), lordosis (4.5%), tendonitis (4.5%)	Anxiety disorder: anxiety (54.5%) Cognitive: cognitive fogs, distress, exhausting, self-regulation (spoon theory), self-doubt, lonely, depressed, worthlessness, self-hate Learning difficulty: dyslexia (4.5%) Mood disorder: depression (68.2%), bipolar disorder (9.1%) Negative affect: frustration, burdensome (symptoms), overwhelming (symptoms), distress; Suicidal ideation: suicidal thoughts Miscellaneous: CRPS (9.1%), chronic fatigue (27.3%), pain, fatigue, iatrogenic physical harms	Education: high school/college dropout Employment: did not pursue careers Healthcare experiences: encountered lack of understanding, encountered lack of empathy, dismissive treatment by healthcare professionals, distrust of medical providers, incomplete diagnoses, aversion to hostile clinical environments Hobbies/daily activities: abandoned hobbies/athletic pursuits due to physical limitations, daily activity limitations Social relationships: creeping isolation, lost entire social groups, lost support of loved ones, unable to participate in activities, relationships atrophied, lonely, social networks crumbling;	Specific, difficult struggles faced, cases are complex. Long, overwhelming struggles of the diagnostic odyssey explains where and how these tensions arise. The journey is both limited and limiting. A significant misconception to think of these patients as difficult. Extraordinary lengths to find a medically valid explanation. Encounters are made difficult by the burden of this long and tumultuous odyssey. Need for greater empathy in the patient–provider relationship
Maya, et al. (2021) [20]	ANS/Cardiology: dysautonomia (70%); postural symptoms (62%); palpitations (53.5%), chest pain/discomfort (33.7%), hypotension (10.9%), exercise intolerance (78%), true/near syncope (37.6%), LE oedema (14.9%) Gastroenterology (61.4%) Respiratory: dyspnoea (35.6%), chest pain/discomfort (33.7%) Urology (8.9%) Neurology: dizziness (70.3%), poor concentration (46.5%) Miscellaneous: Fatigue (77.2%), thermodys regulation (33.7%), pain (50%)	Miscellaneous: fatigue (77.2%), pain (50%), palpitations (53.5%)	Lifestyle: sedentary lifestyle (> 75%)	Having hEDS/HSD or dysautonomia are at risk for substantial morbidity and impaired QoL. Having both hEDS/HSD and dysautonomia imposes a greater symptomatic burden and disproportionately affects young women. hEDS/HSD leads to exercise intolerance, avoidance and a sedentary lifestyle
Wasim, et al. (2019) [21]	Cardiology (41.2%) Dermatology (60.6%): skin hyperextensibility Gastroenterology (61.6%) Haematology (63.2%) MSK/Orthopaedics (96.9%): joint hypermobility, recurrent joint dislocations Neurology (54.5%) Ophthalmology (46.3%) Rheumatology: rheumatoid arthritis, systemic lupus erythematosus Urogenital (24%) Miscellaneous: pain (85.7%)	Anxiety disorder (28.6%) Behavioural disorder (11.5%): ADHD (4.6%) Learning difficulty: dyslexia/speech difficulties (2.8%), self-harm/suicide (0.5%) Mood disorder (34.5%): depression (30.2%), bipolar disorder (1.9%) Personality disorder: schizotypal disorder (0.3%) Psychosis: schizoaffective disorder (0.3%)	NS	Mood and somatoform disorders were associated with pain and GI dysfunction. ADHD was more frequent in HSD. Psychiatric findings and systemic associations were similar across the spectrum of HSD/EDS, management should be similar regardless of diagnosis. Importance of screening for mental health disorders in HSD/EDS, particularly those with pain and GI symptoms. Awareness of the associations will aid management and identify those at a higher risk for mental health disorders
Saetre & Eik (2019) [22]	MSK/Orthopaedics: hypermobile (body capable of unlimited movement), pain as children (dismissed as growth pain), decreased function, bodily disruption, bodily discomfort, body feels loose, body failure, sudden falls, imbalance of body tolerance, range of flexibility Miscellaneous: fatigue, feeling a lack of energy, sleep disturbances	Negative affect: frustration (perceived lack of knowledge and understanding), feeling a lack of energy, overwhelming, anger, grief, distress, fear, hope and hopelessness, uncertainty, disharmony	Hobbies/daily activities: daily activities, lack of energy for activities, exercise-daily activity imbalance, restrictions	Complex experiences of having flexible bodies and restricted lives. Pain, fatigue and weakness demand attention and resolution, causing individual suffering. HCPs need to develop an ethical attitude and sensitivity in listening, and acknowledge bodily experiences to understand the nature of this illness An integrated approach in the management may provide a deeper understanding, better clinical decision‐making and improved possibilities for recovery
Terry, et al. (2015) [23]	MSK/Orthopaedics: recurring joint dislocation Miscellaneous: fatigue, pain (chronic and acute), proprioception	Anxiety disorder: heightened anxiety Cognitive disorder: catastrophising Negative affect: modify/restrict behaviours, stigmatised, fraudulent Miscellaneous: fatigue, pain	Hobbies/daily activities: modify/restrict behaviours, activity pacing	Diverse, fluctuating and debilitating symptoms. Diagnosis is slow, exacerbating symptoms. Increased awareness in primary care could help improve the diagnosis and referral processes. Access to JHS-trained professionals could help patients to effectively manage their condition and receive psychological support as needed. Patients and professionals should learn from one another and assist in developing a deeper understanding of a poorly understood condition
Mullick, et al. (2013) [24]	Dermatology: skin laxity (1.2%) MSK/Orthopaedics: excessive joint clicking and laxity (25%), recurrent joint dislocations (8.33%), carpal tunnel syndrome (1.2%), knee pain (51.2%), low backache (23.8%) Rheumatology: non-specific polyarthralgia (52.38%), soft tissue rheumatism (13.1%), fibromyalgia (4.76%), synovitis (4.76%) Miscellaneous: lateral head tilt (10.71%)	Anxiety disorder (1.2%)	NS	Under-recognised and usually missed in clinical practice due to a lack of awareness. Need to consider even when presenting with non-specific MSK symptoms. The ‘lateral head tilt sign’ was an incidental finding in 10%, could be a leading clue
Martinez, et al. (2021) [25]	ANS/Cardiology: dysautonomia, mitral valve prolapse (0% vs 3.6% vs 25.5%), aortic root dilation (0% vs 0% vs 6.1%) Dermatology: unusually soft or velvety skin (27.3% vs 67.9% vs 79.6%), mild hyperextensibility skin (31.8% vs 50% vs 51%), unexplained striae (27.3% vs 28.6% vs 59.2%), bilateral piezogenic papules (36.4% vs 39.3% vs 73.5%), atrophic scarring (13.6% vs 35.7% vs 55.1%) Gastroenterology: lump in throat, difficulty swallowing, nausea, vomiting, chest pain, intolerance of several foods, abdominal fullness, abdominal bloating, feeling of abdominal distension, abdominal pain, bowel noises, stools very rarely, frequent changing of stool consistency, hard or lumpy stools, straining during bowel movement, passage of mucous, feeling of incomplete evacuation, recurrent/multiple abdominal hernias (13.6% vs 7.1% vs 11.2%) Gynaecology: prolapse (pelvis, rectal, uterine) (0% vs 3.6% vs 15.3%) MSK/Orthopaedics: MSK/CWP (31.2% vs 89.3% vs 92.9%), joint dislocation/instability (9.1% vs 17.9% vs 38.8%), arachnodactyly (9.1% vs 28.6% vs 50%), arm span-to-height ratio (0% vs 3.6% vs 14.3%) Orthodontics: dental crowding/high narrow palate (18.2% vs 5.6% vs 69.4%) Miscellaneous: fatigue, daytime sleepiness	Negative affect: fear of movement Miscellaneous: fatigue	Hobbies/daily activities: fatigue affects activities	HSD/hEDS groups had significant impairment of health-related QoL, complaints of generalised pain and self-reported symptoms including fear of movement, fatigue, daytime sleepiness, gastrointestinal concerns and dysautonomia
Folci & Capsoni (2016) [26]	Dermatology: thin skin, elastic skin Gastroenterology: abdominal discomfort, alternating bowel MSK/Orthopaedics: recurrent sprains, joint pain, pain in the TMJ, dislocate TMJ, twist and stretch thoracic-lumbar spine, axial and peripheral joint mobility, widespread joint hypermobility, extra-range mobility (knees, elbows, fingers) Rheumatology: generalised arthralgia, myalgia, diffuse arthralgia (shoulders, ankles, wrists, knees) Urology: difficult urination, dysuria, pollakiuria Miscellaneous: persistent fatigue, tendonitis (wrists and ankles), lower limb paraesthesia, sleep disturbance, weight gain, chewing problems	Cognitive: poor concentration Mood disorder: depressive mood Negative affect: frustration Miscellaneous: fatigue, sleep disturbance, pain	Daily living: bedridden, change in lifestyle, heavy impact on life Hobbies/daily activities: ceased physical activities	Clinical complexity and the multidisciplinary importance of an unexpectedly common disease that still tends to be under-recognised. Early diagnosis is essential to avoid long and unnecessary diagnostic paths. Management requires a coordinated intervention including patient education, personalised physiotherapy and multidisciplinary medical collaboration
Baeza-Velasco, et al. (2016) [27]	ANS/Cardiology: dysautonomia; tachycardia Dermatology: thin skin, easy bruising, thin and hyper-extensible skin Gastroenterology: constipation, abdominal pain, dysphagia, gastroesophageal reflux, bloating, chronic nausea, food intolerances, painful swallowing, painful digestion, nausea, vomiting Genetics: unequivocally affected first degree relative Haematology: haemorrhages (nasal & gingival) MSK/Orthopaedics: recurrent dislocations, MSK pain, chronic MSK pain, recurrent blocks, recurrent sprains, TMJ dislocation, mild scoliosis Neurology: migraine Respiratory: respiratory dysfunction Rheumatology: chronic arthralgia Miscellaneous: Marfanoid habitus, chronic fatigue, hyperosmia, enhanced audition (hyperacousia), touch sensitivity (cutaneous hyperesthesia), proprioception dysfunction (clumsiness, frequent trips and falls), chronic fatigue, sleep disturbances, lack of appetite, weight loss, amenorrhea, masticatory muscle pain	Anxiety disorder: panic attacks without agoraphobia Eating disorder: eating avoidance, AN, eating behaviours, distorted body image, fear of gaining weight Mood disorder: depression Negative affect: depressive feelings Substance use disorder: cannabis dependence Miscellaneous: pain, fatigue, sleep disturbances, self-injurious behaviours	Education: bullying experiences Substance use: cannabis dependence	Features and common co-occurring problems may favour difficulty eating, significant weight loss and eating disorders such as AN with consequent poor nutrition. The relationship with eating problems warrants more clinical and research attention
Lee and Strand (2017) [28]	Cardiology: tachycardia, syncope, postural hypotension Dermatology: bruise easily, thin skin, scar fissure, atopic, atrophic scars Gastroenterology: bloating, nausea, involuntary vomiting, dysphagia, weight loss, hiatus hernia, mild esophagitis MSK/Orthopaedics: general joint hypermobility, joint pain, dislocations, lumbar lordosis, chronic compartment syndrome, sprains Orthodontics: overbite, dental crowding, high/narrow palate Miscellaneous: diffuse pain	Eating disorder: eating disorder/behaviours Miscellaneous: somatic conditions, pain	NS	Heterogeneous presentation. EDS symptoms may resemble or mask an underlying eating disorder, and vice versa. GI manifestations could be a risk factor for developing disordered eating
Berglund, et al. (2015) [29]	MSK/Orthopaedics: back pain (94%), cervical back pain (82%), thoracic back pain (74%), lumbosacral back pain (81%) Miscellaneous: tiredness	Anxiety disorder: anxiety (74.8%) Mood disorder: depression (22.4%) Miscellaneous: tiredness	Lifestyle: daily life	A lower health-related QoL was found among EDS middle-aged individuals. Probable anxiety and depression were detected. Important to explore the factors behind these results and what initiatives can be taken to alleviate the situation for this group
Clark, et al. (2014) [30]	ANS (70% vs 12%)/Cardiology: light headedness, fainting, dizziness, PoTS (7.8%) Functional difficulties: gross motor activities, fine motor activities, organisation, impaired co-ordination, ability at games, ball skills, impaired balance, obstacle avoidance Gastroenterology (71% vs 9%): nausea, constipation, diarrhoea, stomach ache MSK/Orthopaedics: Knee pain (86%), lower back pain (83%), elbow pain (39%), foot pain (56%) Rheumatology: fibromyalgia (19% vs 0%) Miscellaneous: CFS (31% vs 1%), CWP (86%)	Developmental disorder: DCD/dyspraxia (56% vs 19%); Miscellaneous: pain	NS	Clinicians should assess for broader neurophysiological symptoms in patients presenting with CWP in order to understand and better manage this condition. Adds to the growing body of evidence that recognises the multifactorial manifestations and the need to be recognised and treated holistically. Need to explore effective interventions that ameliorate the debilitating symptoms
Inayet, et al. (2018) [31]	Gastroenterology: abdominal pain (61% vs 28%), diarrhoea (33% vs 9%), constipation (54% vs 17%), centrally mediated abdominal pain syndrome (10% vs 3%), functional biliary pain (1% vs 1%); *Oesophageal disorders:* functional chest pain, (7% vs 1%), functional heartburn (33% vs 11%), globus (3% vs 1%), functional dysphagia (14% vs 3%); *Gastroduodenal disorders:* functional dyspepsia (61% vs 7%), belching disorders (11% vs 4%), nausea and vomiting disorders (4% vs 1%), rumination syndrome (3% vs 1%); *Bowel disorders:* IBS (23% vs 7%), functional constipation (36% vs 11%), functional diarrhoea (21% vs 6%), functional abdominal bloating/distension (26% vs 7%), unspecified functional bowel disorder (56% vs 24%); *Anorectal disorders:* faecal incontinence (2% vs 1%), functional anorectal pain (4% vs 2%)	QoL	QoL	A greater understanding of functional GI symptoms may give further understanding to the aetiology of GI symptoms in individuals not formally diagnosed with connective tissue abnormalities and may give insight into the causes of functional GI disorders and IBS
Albayrak, et al. (2015) [32]	Localised pain: neck (20%), low back (32.2%), knee (27.8%), back (5.2%), ankle (4.3%), wrist (8.7%), other joint (1.7%) Miscellaneous: physical function, role physical, bodily pain, general health, physical component summary, fatigue, sleep quality	Mood disorder: depression QoL: impaired Miscellaneous: bodily pain, role emotional, mental health, mental component summary, vitality, sleep quality, fatigue	Lifestyle: vitality QoL: impaired Social relationships: social function	Increased depression levels, fatigue and diminished QoL are common. One factor may trigger or aggravate another. Assessments should focus on the pain complaint, psychological problems, fatigue, sleep patterns, and QoL. A holistic approach to the examination, assessment, thorough questioning and a multidisciplinary treatment regimen
Gaisl, et al. (2017) [33]	Respiratory: OSA (32% vs 6%), hypopneas (64%), apnoeas (36%) Miscellaneous: fatigue, day time sleepiness	Negative affect: depressive symptoms Miscellaneous: fatigue	QoL: lower	OSA is highly prevalent and under-recognised and contributes to fatigue, daytime sleepiness and impaired QoL in this population. Patients with EDS and excessive daytime sleepiness should be evaluated for OSA
Zeitoun, et al. (2013) [34]	Gastroenterology: heartburn (68.7%) regurgitations (68.7%), decubitus (62.6%), dysphagia (62.6%), epigastric pain (78.8%), nausea (70.8%), postprandial fullness (67.2%), belching (70.5%), IBS (48%), functional constipation (46%) Orthodontics: erosion of dental enamel (51.5%) Respiratory: chronic cough (36.2%), laryngitis (56.8%), asthma (45%)	QoL	QoL	Digestive manifestations are extremely common, most frequently nonspecific and not serious but with major consequences on QoL. A systematic clinical assessment of the EDS population is required. Improve therapeutic management
Fikree, et al. (2017) [35]	ANS/Cardiology: PoTS (60%) Gastroenterology: reflux symptoms, regurgitation, heartburn, dysphagia, NERD (53%), reflux hypersensitivity (21%), functional heartburn (25%), pathological acid reflux, increased acid exposure, hiatus hernia (23%), small hernias (33%), hypotensive LOS (33%), ineffective oesophageal motility (40%)	Anxiety disorder Mood disorder: depression	NS	A large proportion of JHS patients with oesophageal symptoms have true reflux related symptoms and oesophageal dysmotility. There is an over-representation of reflux hypersensitivity and oesophageal hypomotility. More likely if JHS patients have comorbid PoTS
Mastoroudes, et al. (2012) [36]	Gynaecology: heaviness/dragging (28.3% vs 5%), discomfort (25% vs 5%), sex interference (bulge) (27% vs 10%), bowel interference (bulge) (23% vs 5%), straining to open bowels (61.7%), posterior compartment prolapse, bowels incompletely emptying MSK/Orthopaedics: backache (60% vs 23.3%), lower backache (36.7% vs 5%)	QoL	QoL Social relationships: sex interference (bulge) (27% vs 10%)	The prolapse impacts QoL in the form of bowel evacuation symptoms and sexual dysfunction. The incidence of prolapse and the symptoms is significant, much may still go unnoticed. Provides knowledge about the pathophysiology of prolapse and obstructed defecation
Mastoroudes, et al. (2013) [37]	Gynaecology/Urology: urinary incontinence (73.3% vs 48.3%), urgency incontinence (62% vs 38.3%), stress incontinence (63.3% vs 36.7%) voiding dysfunction (63.3% vs 23.3%), straining to empty bladder (48.3% vs 13.3%), poor stream (38.3% vs 8.3%), postmicturition dribble (46.7% vs 30%), anterior prolapse, nocturia, nocturnal enuresis, intercourse incontinence, bladder pain	Negative affect: embarrassment QoL	QoL	Incontinence is a source of embarrassment. Clinicians rarely acknowledge the impact it has on QoL. A large proportion of time is spent in rheumatology and hypermobility clinics with an insignificant amount of time spent asking about gynaecological issues. Under-diagnosis may be due to underreporting of symptoms or failure by GPs and rheumatologists to routinely screen for these symptoms. A systematic approach may be more effective in identifying these cases. A high prevalence of incontinence justifies the need for an integrated continence pathway within specialised hypermobility units
Puledda, et al. (2015) [38]	ANS/Cardiology: orthostatic hypotension (36.3%), PoTS (15.2%), mitral valve collapse (21.1%), arrhythmias (9.1%) Dermatology: psoriasis (12.1%) Gastroenterology: GORD (57.6%), chronic gastritis (30%), hiatal hernia (12.1%), celiac disease (9%) Gynaecology: urogynaecological prolapses and stress incontinence (12.1%) MSK/Orthopaedics: TMJD (18.2%) Neurology: *Migraines:* pulsating pain (75.8% vs 80.3%), constrictive pain (24.2% vs 19.7%), unilateral pain (36.4% vs 37.9%), bilateral pain (36.4% vs 39.4%), bilateral and unilateral (27.3% vs 22.7%), photophobia (100% vs 89.4%), phonophobia (78.8% vs 94%), osmophobia (54.6% vs 41%), nausea (91% vs 80.3%), vomiting (48.5% vs 48.5%), visual aura (36.4% vs 39.4%), sensory aura (3% vs 4.54%), speech aura (3% vs 1.5%) Respiratory: allergic asthma (15.2%) Rheumatology: Raynaud’s phenomenon (18.2%) Miscellaneous: multiple medication allergies (27.3%), inner ear dysfunction (18.2%), ANA positivity (6%)	Anxiety disorder: anxiety with panic attacks (51.2%) Mood disorder: mood disturbances (36.4%), bipolar disorder (6%) Psychosis: psychotic disorder (3%)	NS	Migraine has a high impact on QoL in these patients. Although associated it must always be recognised separately and not contemplated solely as one of the manifestations of the disease. Adequate migraine therapy is essential in these patients and should not be delayed, to allow a lower risk of migraine-associated disability
Bulbena-Cabre, et al. (2018) [39]	NS	Anxiety disorder (16.7%): GAD (7.4%), panic (3.7%), agoraphobia (3.7%), social phobia (2.8%), specific phobia (2.8%); Anxiety with comorbid depression (34.8% vs 11.8%), depression with comorbid anxiety (30.4% vs 10.6%) Mood disorder (14.8%): major depression (8.3%), dysthymia (6.48%)	NS	JHS is strongly associated with anxiety disorders in the elderly population
Baeza-Velasco, et al. (2022) [40]	Severe usual pain (53.1%)	Anxiety disorder (45.7%): traumatic event (42.9%) Eating disorder (17.1%) Mood disorder: major depression (37.1%), maniac/hypomanic episode (28.6%) Substance use disorder: alcohol/substance abuse (28.6%) Suicidal ideation: suicidal attempt (31.4%)	Substance use: alcohol/substance misuse	Additional burden of psychiatric comorbidity. A more systematic screening of psychological variables (e.g., anxiety and personality disturbances) should be done in individuals presenting with hEDS
Liaghat, et al. (2022) [41]	MSK/Orthopaedics: shoulder dislocation (18%), shoulder feeling loose (48%), cracking, clicking, snapping, instability, looseness, pain, strength, range of movement, proprioception, discomfort Miscellaneous: fatigue	Negative affect: fear of movement QoL Miscellaneous: fatigue, pain	Employment Hobbies/daily activity: sports, recreation QoL	HSD and shoulder complaints present with impairments related to shoulder pain, function, fatigue, fear of movement, and QoL. Self-reported clinical characteristics were more severe for those with mechanical symptoms, longer symptom duration, shoulder dislocations, feeling the shoulder is loose, and additional discomfort, received supplemental treatment. Importance of addressing mechanical symptoms in the shoulder during treatment to fully cover and understand the patients’ impairments
Berglund & Björck (2012) [42]	Miscellaneous: mucosal problems, oral problems, nasal problems, eye problems, genital problems, physical pain, handicap, functional limitation	Negative affect: felt insecure, felt tense, difficulty relaxing, felt embarrassed, life has been less satisfactory, psychologic discomfort	Lifestyle: life has been less satisfactory	Women with EDS report a low oral health related QoL as measured with the OHiP-14. Particularly in physical pain, psychologic discomfort, and handicap. This demonstrates that also the oral health-related QoL is impeded by the disorder
De Baets, et al. (2021) [43]	MSK/Orthopaedics: physical discomfort, dislocation, overload injury, joint pain (hands, shoulders, back, neck), muscle cramps Miscellaneous: pain, heavy, tiring, fatigue, energy	Negative affect: stigma, self-esteem (value in society), heavy Miscellaneous: pain, fatigue, energy	Employment: economic factors (financial independence), work absence, lack of job opportunities, lacking of resources and information and support to adapt working environment, unreliability, commuting obstacles, work-life imbalance, job performance, accessibility of the workplace Social relationships: social isolation	Health-related complaints, pain, fatigue, the imbalance between having a chronic disease, private life and work, determined the level of work participation. Participating in work contributes to the well-being and has positive health consequences. Need to udnerstand of the needs of people with hEDS and their participation in working life
Nee, et al. (2019) [44]	Gastroenterology: *Functional gastrointestinal disorder (FGIDs):* IBS (57.8% vs 27%), IBS-C (13.4% vs 7.5%), IBS-D (11.8% vs 5.5%), dyspepsia (55.4% vs 25%), postprandial distress (49.9% vs 21.7%), epigastric pain (0.1% vs 0.2%), functional constipation (7.3% vs 5.3%), functional diarrhoea (0.7% vs 1.5%), heartburn (33.1% vs 16.8%), chest pain (2.1% vs 1%), dysphagia (28.5% vs 18.3%), globus (2.3% vs 1.2%), CVS (20.6% vs 10%), rumination (5.1% vs 1.7%), bloating (12.4% vs 16.3%), aerophagia (24.7% vs 12.3%), chronic idiopathic nausea (24.7% vs 7.2%), functional vomiting (4% vs 1.5%); Gynaecology: *Pelvic floor symptoms:* haemorrhoids (59.2% vs 42.5%), anal fissure (47.7% vs 20.5%), rectal prolapse (16.4% vs 6%), faecal incontinence (19.2% vs 11.2%), incomplete evacuation (83.3% vs 65.8%), suggestive of functional defecation (60.2% vs 34.5%), chronic proctalgia (14.3% vs 5.3%), proctalgia fugax (24.7% vs 14.7%), urinary incontinence (60% vs 38.8%), incomplete urinary voiding (75.3% vs 50.5%), hysterectomy for bleeding (13.5% vs 8.2%), uterine prolapse (13.1% vs 5.8%), endometriosis (24.1% vs 10.2%), rectocele (14.3% vs 3.7%)	Miscellaneous: unhealthy mental health days	QoL	Prevalence of some FGIDs in MFS is high, similar to the prevalence in the general US population. EDS were significantly more likely to suffer from FGIDs compared MFS and the prevalence was higher than in the general population. Pelvic floor symptoms were common in EDS and MFS, but more common in EDS compared to MFS
Bisaralli, et al. (2017) [45]	Cardiology: palpitations MSK/Orthopaedics: hyperextension (elbow), pain (shoulder, knees and elbows), burning sensation in feet Miscellaneous: weight loss, decreased appetite, Marfanoid habitus, oral ulcers	Miscellaneous: pain, palpitations	NS	Four times higher risk of anxiety, depression, and panic disorders. Could be detected in day‐to‐day practice, diagnosed and managed properly. Advantages in some careers (e.g., gymnastics) but serious MSK consequences (e.g., recurrent joint dislocations and premature osteoarthritis). Effective treatment may include advising proper body mechanics and conferring the joint protection

*ADHD* attention deficit hyperactivity disorder; *AN* anorexia nervosa; *ANS* autonomic nervous system; *BJHS* benign joint hypermobility syndrome; *BPD* borderline personality disorder; *CFS* chronic fatigue syndrome; *CRPS* chronic regional pain syndrome; *CVS* cyclic vomiting syndrome; *CWP* chronic widespread pain; *DCD* developmental co-ordination disorder; *EDS* Ehlers-Danlos syndrome; *EDS-HT* Ehlers-Danlos syndrome hypermobility type; *FGIDs* functional gastrointestinal disorders; *GAD* generalised anxiety disorder; *GERD/GORD* gastro-oesophageal reflux disease; *GI* gastrointestinal; *GPs* General Practitioners; *HCPs* healthcare professionals; *hEDS* hypermobility Ehlers-Danlos syndrome; *HSD* hypermobility spectrum disorders; *IBS* irritable bowel syndrome; *IBS-C* irritable bowel syndrome with constipation; *IBS-D* irritable bowel syndrome with diarrhoea; *IBS-M* irritable bowel syndrome mixed; *IBS-U* irritable bowel syndrome undefined; *JHS* joint hypermobility syndrome; *LOS* lower oesophageal sphincter; *MCAS* mast cell activation syndrome; *MFS* Marfans syndrome; *MS* multiple sclerosis; *MSK* musculoskeletal; *NERD* non-erosive reflux disease; *NS* not stated; *OSA* obstructive sleep apnoea; *PCOS* polycystic ovary syndrome; *PoTS* postural tachycardia syndrome; *PTSD* post-traumatic stress disorder; *QoL* quality of life; *TMJ* temporomandibular joint; *TMJD* temporomandibular joint dysfunction; *US* United States

**Table 4:** HCP involvement, measurements used, management/treatment referenced

Author (year)	HCP involvement	Measurements	Management/treatment
Palomo-Toucedo, et al. (2020) [6]	NS	NS	Medication; resting; support groups
Hershenfeld, et al. (2016) [9]	Genetic clinics	NS	NS
Alomari, et al. (2020) [16]	Cardiologist; Gastroenterologist; Geneticist (diagnosis); Rheumatologist (diagnosis)	Gastroenterology: Rome III criteria; Rome IV criteria HSD/EDS: Brighton criteria; Villefranche nosology; 2017 international criteria for hEDS	Gastric emptying; neuroleptic/antipsychotic use (15.6%); opioid use (37.5%); parenteral nutrition; prokinetic use (31.2%); surgical intervention; tube feeding
Baeza-Velasco, et al. (2018) [17]	EDS unit; National expert practitioner in EDS	Anxiety and depression: Hospitals and Anxiety Scale (HADS) Pain: pain catastrophising scale (PCS); Pain verbal rating scale; Tampa Scale Kinesiophobia (TSK) QoL: Short Form Survey (SF-36) Social support: Social support questionnaire (SSQ-6) Somatoform: Somatosensory amplification scale (SSAS)	Antidepressant medication; Anxiolytics
Murray, et al. (2013) [18]	Genetics; Orthopaedics; Paediatrics; Pain clinic; Physical medicine; Primary care; Rheumatology	Depression: Centre for Epidemiologic Studies Depression Scale (CES-D) Pain: McGill pain questionnaire	Antidepressant medication
Halverson, et al. (2021) [19]	Acupuncturist; Chiropractor; Generalists; Massage therapist; Neurologist; Physical therapists; Psychologists; Specialists	NS	Exercises; surgeries; wheelchair
Maya, et al. (2021) [20]	Cardiologists; Neurologists; Orthopaedists; Pain management specialists; Primary care providers; Rheumatologists	HSD/EDS: 2017 international criteria for hEDS; Beighton score	Physical therapy program
Wasim, et al. (2019) [21]	Genetic medicine	HSD/EDS: 2017 international classification criteria for hEDS; Villefranche criteria	NS
Saetre & Eik (2019) [22]	NS	NS	Massage; meditation; pacing; relaxation; rest; sleep; training
Terry, et al. (2015) [23]	MSK specialist; Physiotherapists	NS	Physiotherapy; self-management
Mullick, et al. (2013) [24]	Cardiac; Dermatological; Ophthalmic; Rheumatology	HSD and EDS: Beighton score; Brighton criteria	NS
Martinez, et al. (2021) [25]	NS	Autonomic: Composite Autonomic Symptom Score (COMPASS-31); Autoimmune Dysautonomia Evaluation Panel Fatigue: Fatigue Severity Scale (FSS); Epworth Sleepiness Scale (ESS) Gastroenterology: Gastro questionnaire Pain: TSK QoL: SF-36 Somatoform disorders: Patient Health Questionnaire (PHQ-15)	NS
Folci & Capsoni (2016) [26]	Neurologist; Podiatrist; Rheumatologist; Specialised hypermobility	HSD/EDS: Brighton criteria; Beighton score	Medications; pain management; physical therapy (regaining muscle tone and improving proprioception); Podiatry
Baeza-Velasco, et al. (2016) [27]	Neuropsychiatry; Psychiatry	Body image: Contour Drawing Rating Scale (CDRS) HSD/EDS: Brighton criteria; Beighton score Neuropsychiatric: Mini international neuropsychiatric interview (MINI-DSM-IV)	Rehabilitation service
Lee & Strand (2017) [28]	Gastroenterologist; GP; Physiotherapist; Rheumatologist	NS	CBT; knee orthosis; meclizine & ondansetron; pain medication; specialised physiotherapy
Berglund, et al. (2015) [29]	NS	Anxiety and depression: HADS QoL: SF-36	NS
Clark, et al. (2014) [30]	Hypermobility clinic; Rheumatologist	Functional difficulties: Functional Difficulties Questionnaire-9 (FDQ-9) HSD/EDS: Brighton criteria; Beighton score	NS
Inayet, et al. (2018) [31]	NS	Gastroenterology: Rome IV diagnostic criteia HSD/EDS: Beighton criteria QoL: SF-36	Gastrointestinal medications
Albayrak, et al. (2015) [32]	NS	Depression: Beck Depression Inventory (BDI) Fatigue: Checklist Individual Strength (CIS), Pittsburgh Sleep Quality Index (PSQI) HSD/EDS: Brighton criteria Pain: Visual Analog Scale (VAS) QoL: SF-36	NS
Gaisl, et al. (2017) [33]	Respiratory	Depression: Patient Health Questionnaire-9 (PHQ-9) Fatigue: ESS; PSQI HSD/EDS: Villefranche criteria; Beighton score	NS
Zeitoun, et al. (2013) [34]	National expert practitioner in EDS	Gastroenterology: Rome III; Gastrointestinal QoL index (GIQLI) HSD/EDS: Villefranche criteria; Beighton Scale	NS
Fikree, et al. (2017) [35]	Gastrointestinal; Rheumatologist	Anxiety and depression: HADS Gastroenterology: Reflux Disease Questionnaire (RDQ)	Antidepressants with anticholinergic effects; Opiates
Mastoroudes, et al. (2012) [36]	Gynaecological; Hypermobility clinics	Gynaecology: Pelvic organ prolapse/urinary incontinence sexual questionnaire (PISQ-12); Prolapse QoL questionnaire (P-QOL); Pelvic organ prolapse quantification (POP-Q) HSD/EDS: Brighton Criteria; Beighton score	NS
Mastoroudes, et al. (2013) [37]	Gynaecology; Hypermobility clinics; Rheumatology	Gynaecology: P-QOL; POP-Q QoL: King’s Health Questionnaire (KHQ);	NS
Puledda, et al. (2015) [38]	Heritable connective tissue disorder clinic; Neurology	HSD/EDS: Villefranche criteria; Brighton Criteria Migraine and headache: Migraine disability assessment (MIDAS); Headache Impact Test-6 (HIT-6); 3 item Migraine questionnaire Pain: Numeric Rating Scale (NRS)	AEDs; analgesics; anti-depressants; B-blockers; calcium antagonists; NSAIDS; triptans
Bulbena-Cabre, et al. (2018) [39]	Psychiatrist	Anxiety: Anxiety Inventory (STAI) Fear: Fear Survey Schedule (FSS) General health: General Health Questionnaire-28 HSD/EDS: Hospital del Mar criteria	Psychotropic drugs
Baeza-Velasco, et al. (2022) [40]	Expert in EDS	Neuropsychiatric: Mini international neuropsychiatric interview Pain: 5 item verbal rating scale pain Personality disorders: Personality Diagnostic Questionnaire 4 (PDQ-4) QoL: SF-36 Resilience: Connor Davidson Resilience Scale 10 item (CD-RISC 10) Social support: SSQ-6 Stress and coping: The Ways of Coping Checklist (WCCL)	NS
Liaghat, et al. (2022) [41]	Medical clinics; Physiotherapists	Fatigue: CIS HSD/EDS: Five-part questionnaire (5PQ) Pain: TSK QoL: EQ-5D-5L Shoulder instability: Western Ontario Shoulder Instability Index (WOSI)	Chiropractic; injection; medication; physiotherapy; surgery
Berglund & Björck (2012) [42]	NS	Oral health: Oral Health Impact Profile 14 (OHIP-14)	NS
De Baets, et al. (2021) [43]	Physiotherapist	NS	NS
Nee, et al. (2019) [44]	NS	Gastroenterology: Rome III diagnostic questionnaire QoL: QoL; Health Related QoL	NS
Bisaralli, et al. (2017) [45]	Physical medicine; Rehabilitation specialists	HSD/EDS: Brighton criteria; Beighton score	Rehabilitation

*AEDs* anti-epileptic drugs; *CBT* cognitive behavioural therapy; *EDS* Ehlers-Danlos syndrome; *GP* General Practitioner; *HCP* healthcare professional; *hEDS* hypermobility Ehlers-Danlos syndrome; *HSD* hypermobility spectrum disorders; *QoL* quality of life; *MSK* musculoskeletal; *NS* not stated; *NSAIDS* non-steroidal anti-inflammatory drugs

#### MSK and dermatology

Limb hyperextension, joint flexibility, pain and dislocations are the most recognised symptoms of EDS and HSD, frequently reported throughout studies. One qualitative study [23] described how these MSK symptoms, like having a flexible body, can cause restrictions to daily living. Another [24] described how involvement from a specialist hypermobility physiotherapist improved their understanding of their own bodies via a two-way learning process which in turn motivated these individuals to engage in appropriate self-management of their condition. Within assessments of EDS/HSD, individuals are subject to dermatological evaluation [25], this is because skin hyperextensibility is as common as MSK symptoms [9, 26] with additional dermatological symptoms identified such as, thin skin [27–29] and abnormal scarring [9, 19]. A significant number of participants in one study [19] suggested that the MSK and skin symptoms began as early as childhood yet despite these widely recognised and long-standing symptoms, the condition is often underdiagnosed [25].

#### Pain and fatigue

Pain management specialists [21] can refer patients to be evaluated for a joint hypermobility condition due to the high prevalence of pain reported by this population. The pain experienced by these individuals affects the entire body including, but not limited to, back (94%) [30], knee (86%) [31], abdominal (61%) [32], foot (56%) [31], elbow (39%) [31] and neck (20%) [33]. The intensity of the pain ranges from, tiring and exhausting (93%) [19], chronic and constant (67%) [19] and severe to very severe (66%) [18]. Prevalence of fatigue is also reported to be as high as 77% [21] and can be measured using the Epworth Sleepiness Scale (ESS) [26, 34] or Fatigue Severity Scale (FSS) [25]. Fatigue and impaired sleep quality are considered to be a simultaneous experience to pain, as described by individuals with EDS/HSD, interfering with their ability to live a good QoL [23, 33]. Interestingly, some studies suggested that the presence of pain increases the likelihood of an individual with EDS/HDS also having a comorbid psychiatric disorder [9, 22].

#### Gastroenterology

Gastrointestinal symptoms are extremely common in this population, reported in over half of the included studies, therefore involvement from a gastroenterologist is not unusual. One retrospective study found that over 60% of their sample disclosed at least one gastrointestinal symptom at the time of their hEDS diagnosis [17]. Abdominal pain (50%) and nausea (50%) in this sample were the two most common gastrointestinal symptoms [17]. This was supported by another study, reporting higher prevalence found in an EDS sample, 79% and 71% respectively [35]. Furthermore, one study with a hEDS sample and accompanying gastrointestinal symptoms were significantly more likely to use medications for these symptoms (anti-secretory, antacids, laxatives) than the control group (*p* < 0.01) [32]. Though these symptoms are not considered serious, they can cause a detrimental impact to the individual’s QoL, as assessed by the Gastrointestinal Quality of Life Index (GIQLI) [35].

#### Autonomic nervous system (ANS) and cardiology

Dysautonomia is condition characterised by a malfunction of the ANS with associated symptoms such as dizziness, fainting and light headedness [31]. One study [21] found prevalence of dysautonomia at 70% amongst a hEDS/HSD sample, with those carrying this diagnosis significantly more likely to suffer with related symptoms, have an impaired QoL and exhibit exercise avoidance behaviours, subsequently leading to a more sedentary lifestyle. Individuals with dysautonomia and attempting to engage in physical therapy programmes are limited by the related symptoms, leading to a lack of significant improvement in their overall condition via such interventions [21]. This ANS malfunction is also responsible for postural tachycardia syndrome (PoTS), with prevalence in individuals with EDS/JHS ranging from 8% [31] to 64% [20]. Those with both JHS and PoTS were found to be at a significantly increased risk of gastrointestinal symptomology (*p* ≤ 0.05) [17], with higher reflux scores, regurgitation and dysphagia [36].

#### Rheumatology

Fibromyalgia, a chronic widespread pain (CWP) condition, has been identified in a small number of the included studies as a comorbid condition. Prevalence of the diagnosis ranges from as small as 5% [25] up to 41% [20] amongst benign JHS (BJHS) and EDS samples respectively. Interestingly, individuals with hEDS were significantly more likely to report this comorbid diagnosis if they likewise presented with gastrointestinal manifestations and chronic pain (*p* < 0.02) [17]. Complaints of tiring and exhausting pain were further found to be a predictive factor of a fibromyalgia diagnosis within one hEDS sample [19]. Individuals with joint hypermobility conditions have overlapping symptoms as those with fibromyalgia [31], therefore the increased likelihood of also having this CWP condition could be attributed to this reason.

#### Gynaecology and urology

Women are much more likely to be affected by this condition and as a result endure clinically significant urogynaecology-related symptoms like, pelvic organ prolapse (*p* < 0.01) [37, 38] as an example. These symptoms can have a detrimental impact on QoL, as measured by the Prolapse Quality of Life (P-QOL) [37, 38]. The impact of gynaecological issues is rarely acknowledged by the rheumatologists and primary care practitioners who predominantly make the diagnosis [38]. One study [37] found prolapse symptoms to be clinically significant across the general health perception, physical limitation, social limitation, personal relationships, emotions, sleep/energy and severity domains (*p* ≤ 0.05), as well as contributing to sexual and bowel dysfunctions. In addition to this, urinary incontinence was also cited as a clinically significant symptom within a BJHS sample (*p* < 0.01) with prevalence over 70% and a cause of significant embarrassment to women [38].

#### Neurology

Neurological assessments conducted by neurologists found manifestations in this population to include migraines and headaches, with severity of symptoms measured by the Migraine Disability Assessment (MIDAS) and HIT-6 [39]. Individuals with hEDS and migraines are significantly more likely to have an earlier onset of symptoms than those without hEDS (13 vs 17 years, *p* < 0.01), a higher number of days per month migraine episodes (15 vs 9 days, *p* = 0.01) and experience photophobia (*p* = 0.05) [39]. The migraine-related pain hEDS experience has been described as pulsating (76%) and constrictive (24%), with these individuals being significantly more likely to use migraine-related medication (e.g., NSAIDS, analgesics, triptans) (*p* ≤ 0.05) [39]. This is an example of the substantial impact of migraines on the QoL of hEDS individuals. However, it is still recommended that migraines are seen and managed as a separate condition rather than a sole manifestation of hEDS [39].

#### Respiratory

Respiratory symptoms have been inconsistently reported throughout the included studies and are quite varied, suggesting a weak association between EDS and/or HSD individuals. However, one study [34] recognises obstructive sleep apnoea (OSA) as highly prevalent yet under-recognised in HSD when compared to control groups (32% vs 6%, *p* < 0.01), hypopneas being the most common respiratory event within this HSD sample (64%) [34]. It may be that HSD individuals suffering from excessive daytime sleepiness and fatigue should also be assessed for OSA as a potential cause [34].

#### Mood and anxiety disorders

Almost half of included studies suggested EDS/HSD individuals were significantly more likely to experience mood and anxiety disorders than the general population (*p* < 0.01) [22]. The most frequently cited mood disorder being depression with prevalence ranging from 22% [30] to 69% [19], with anxiety having a higher prevalence at 75% [30]. The two disorders were most often measured in studies using the Hospital Anxiety and Depression Scale (HADS) [30, 40], whilst antidepressant and anxiolytic use was also noted within this sample [18]. Additional diagnoses of both mood and anxiety disorders included: major depression (ranging from 8% [41] to 37% [41]), bipolar disorder (less than 10% [20]), phobias [40] and post-traumatic stress disorder (PTSD) [9], though the latter were reported in less than 5%. Notably, one study found that HSD individuals experiencing muscle and body pain and gastrointestinal symptoms were at a significantly increased risk of experiencing depression (*p* < 0.01 and *p* < 0.03 respectively), with gastrointestinal symptoms alone being significant for anxiety (*p* < 0.01) [22].

#### Negative affect and cognition

Negative affect is the experience of negative emotions and psychological distress. This population described being fearful, specifically fear of movement [18, 26, 42]. One study utilised the Tampa Kinesiophobia Scale (TSK) to measure this, finding a statistically significant difference between individuals with EDS/HSD and without, including within the activity avoidance and somatic focus subscales (*p* < 0.01) [26]. Additionally, catastrophising (a cognitive distortion) was cited across two included studies. A sample of hEDS individuals with high levels of anxiety were found to score significantly higher in pain catastrophising (*p* < 0.01), as measured by the Pain Catastrophizing Scale (PCS) [18]. A qualitative study further offered an explanation to the above findings, describing how prior experiences of injuries have led to heightened levels of anxiety, resulting in participants catastrophising about future injuries [24]. The anxiety, fear of movement and pain catastrophising is a direct response to their unpredictable symptoms, resulting in individuals modifying or restricting their activities to avoid pain and potential injuries [26].

#### Eating disorders

Three case studies of young female women described EDS/JHS manifestations that contributed to the development of eating disturbances [28, 29]. Such disturbances included painful eating experiences [43] and selective eating behaviours due to temporomandibular pain and dislocations [28], as well as eating avoidance due to gastrointestinal problems like nausea and vomiting [28]. One female was diagnosed with the eating disorder anorexia nervosa (AN) and underwent an enhanced cognitive behavioural therapy programme to improve eating behaviours, body image and social functioning [29]. Notably, this study is the only that referenced a CBT programme to be utilised within this population, though specifically for an eating disorder. Additionally, their presenting symptoms which had primarily been attributed to AN could also be attributed to the EDS, specifically gastrointestinal symptoms which caused disordered eating [29].

#### Behavioural disorders

Three of the studies reported a comorbid diagnosis of attention deficit hyperactivity disorder (ADHD). One study found this behavioural disorder to be more frequent in EDS/HSD individuals than in the general population (5% vs 3%), though only significantly higher within those with HSD (*p* < 0.01) [22]. The other two studies reported a slightly higher prevalence of ADHD in EDS and hEDS samples, 7% [9] and 11% [17] respectively. The association between ADHD and EDS/HSD however, is weak and unclear and would warrant further exploration.

#### Psychosis, personality disorder and suicidal ideations

Including the psychiatric disorders already outlined, prevalence of others, though relatively low, have also been recognised such as, schizoaffective disorder (0.3% [22] and 1% [9]), schizotypal disorder (0.3% [22] and 1% [9]) and borderline personality disorder (4%) [9]. One study [41] examined suicidal behaviours in women with HSD finding that 31% of the sample had previously attempted suicide whilst 60% presented with a mild suicidal risk. Those with a history of suicidal attempt were also significantly more likely to have personality disturbances (*p* < 0.01), major depression (*p* < 0.03) and anxiety disorders (*p* < 0.04) [41].

#### Education and employment

The education of EDS/HSD individuals is significantly impacted, with individuals dropping out of education [20], being unable to enrol full-time [19] and when in school being subjected to bullying due to their physical appearance [28]. Similarly, these individuals also face difficulties in their professional lives, feeling unable to pursue their desired careers [20] or having to change their current role and handle less responsibilities [19]. These individuals additionally need to consider the accessibility of their workplace and their working environment to ensure it meets their needs and capabilities [44]. Changes to the individuals desired career path and working environment can contribute to reduced job satisfaction and feeling a lack of financial independence [44].

#### Hobbies and daily activities

Individuals with EDS/HSD are often forced to abandon their favourite hobbies or quit their athletic pursuits as a result of the many physical limitations that accompany the condition [20]. Though this is not applicable to all, some instead choose to persevere and make modifications appropriate to what they are capable of achieving and to pace themselves [24]. Whilst others simply lacked the energy, as well as the physical limitations, to participate in recreational activities and activities of daily living, such as doing housework [23]. They would often have to evaluate their energy levels and how they should distribute their energy throughout the day in order to complete simple daily household tasks (e.g., making food and going shopping) and fulfil their personal care needs (e.g., taking a shower) [22].

#### Social relationships

A qualitative study [6] examining the psychosocial influence in the daily life of individuals with EDS demonstrates the difficulties in maintaining social networks as a result of the debilitating symptoms that accompany the condition. They identified social isolation behaviours in some individuals due to not being able to participate in certain activities and the inability to make long-term plans. In order to be able to attend social events, these individuals must plan rest days in advance to conserve their energy, as previously discussed [22]. However, due to these behaviours and adjustments, these individuals often feel like they must justify themselves to friends and family who, like many HCPs, lack proper understanding and insight into the condition.

#### Miscellaneous associations and HCPs

Referrals for joint hypermobility assessments can also be made by cardiologists and orthopaedists [21]. Additional physical and psychological manifestations inconsistently cited across the 32 studies but noteworthy included: haematology [9, 22, 28], orthodontics [26, 29, 35] (e.g., dental crowding), ophthalmology [20, 22] (e.g., glaucoma), endocrinology [20], genetics [28], immunology [20], infectious disease [20], oncology [20], functional difficulties (e.g., DCD/dyspraxia) [31], learning difficulties (e.g., dyslexia [20, 22]), developmental disorders (e.g., Asperger disorder [9]) and substance use disorder (e.g., alcohol/substance use abuse [41]). Individuals with EDS/HSD also engage in complementary therapies such as acupuncture and massage [20].

## Discussion

Of the 182 unique records identified, 32 studies met the inclusion criteria and informed the results discussed within this report. All included studies strongly evidenced the biopsychosocial impact of EDS and HSD on adults living with these conditions, supporting their presenting complaints to be diverse and fluctuating. Associations with some comorbidities and symptoms were found to be more significant than others, ranging in severity from person to person, all the while still demonstrating a detrimental impact to an individual’s QoL. In this review, the findings were separated into sub-specialities of physical (e.g., MSK and dermatology), psychological (e.g., negative affect and cognition) and social categories (e.g., social networks). Relationships between the sub-specialities were referenced throughout. Gastroenterology, mood disorders and anxiety disorders were evidently the most common physical and psychological manifestations of EDS/HSD with obvious implications to QoL cited across over half of the included studies.

Gastrointestinal symptoms are seemingly underdiagnosed across EDS/HSD populations despite its documented high prevalence [17, 35], though the underlying relationship is unclear [45]. These symptoms were also more likely if the individual had comorbid PoTS [9, 17], a condition knowingly linked to EDS/HSD. Similarly, having gastrointestinal symptoms increased the likelihood of individuals being diagnosed with a psychiatric disorder [22]. Eating disorders, like AN, are often diagnosed following the development of disordered eating behaviours as a result of gastrointestinal manifestations, such as nausea and bloating [29]. However, symptoms of EDS/HSD may resemble or even disguise themselves as an eating disorder and vice versa [29].

Amongst psychiatric disorders, anxiety and mood disorders (e.g., depression) were supported to be significantly more frequent in this population compared to the general population. One study argued that these disorders are a major feature of EDS and often these individuals are stigmatised by clinicians for having a mental health disorder without further investigation for the underlying cause and accompanying systemic issues they present with [9]. Pain tends to be the focus for EDS/HSD individuals as one of the predominant clinical symptoms of the condition [33], as well as the associated MSK symptoms. However, it has been found that an individual with pain was more likely to have a psychiatric disorder [9], whilst another study argued depression levels to be as equally common as pain amongst this population [33]. It is clearly not as simple as diagnosing and treating symptoms of EDS/HSD in isolation, but would rather benefit from a holistic approach given that one symptom has the potential to trigger or aggravate another [33].

The review further found, specifically within the five qualitative studies, that individuals with EDS/HSD face difficulties in achieving this diagnosis, frequently describing negative healthcare experiences. One qualitative study in particular provided valuable insight into the diagnostic odyssey of EDS specifically [20]. As a result of these cases being clinically complex and multisystemic, individuals endure overwhelming struggles within the healthcare system in an attempt to find a medically valid explanation for their symptoms. Most often, individuals are misdiagnosed and mistreated, this itself could exacerbate the already debilitating symptoms [24]. An early diagnosis is vital for the individual, to feel psychologically validated, to ensure access to appropriate treatment and to improve health and wellbeing [27].

### Implications for practice

Based on the findings of the scoping review, EDS/HSD is evidently clinically complex and unexpectedly common, yet often goes undetected in daily practices [27, 46]. There is a clear need for a multidisciplinary collaboration to improve awareness, the diagnostic pathways and referral processes for this condition and its associated symptoms which could lead to an earlier diagnosis and better co-ordination of care.

There appears to be less focus within clinical practice for individuals with EDS/HSD that present with non-MSK and dermatological symptoms, such as gynaecological issues [37, 38], gastrointestinal manifestations [32, 35], excessive daytime sleepiness [34], and migraines [39]. This is largely attributed to a lack of training and education amongst HCPs and the clinical complexity of individual cases [20]. Development of a rigorous clinical assessment for EDS/HSD, recognising the multifactorial manifestations, would aid clinicians in making an earlier diagnosis and therefore enable better management of the condition, including referrals to appropriate HCPs as necessary. Such an assessment, for example, would better recognise that individuals with EDS/HSD presenting with both comorbid pain and gastrointestinal symptoms are at a higher risk of suffering from a mental health disorder [9, 22], offering the opportunity for earlier intervention.

Developing an effective assessment and treatment pathway for this condition will inevitably be complex given the diverse range of symptoms and conditions presented in this review. Some studies referenced specialist hypermobility clinics [31, 37, 38] and expert practitioners in EDS [18], though there are only a small number of specialist clinics in the UK [37], not everyone with this condition will have access. Improving the training and education for HCPs could improve the current long and often unnecessary diagnostic pathways these individuals are subjected to. Within this training, ensuring an empathetic patient–provider relationship is key as well as developing an ethical attitude and sensitivity in listening to strengthen the therapeutic relationship and develop a deeper understanding of this misunderstood condition [20, 21, 23].

### Limitations

The review is limited to the adult population as specified by the eligibility criteria. However, it was clear across the included studies that some of these symptoms began before the age of 18 years. The majority of individuals in one study reported hypermobility, joint pain, dislocations and dermatological symptoms to have begun in childhood whilst the onset of depression, anxiety and fatigue mainly occurred after the age of 18 years [19]. The latter could however be attributed to a lack of awareness amongst HCPs of these symptoms being linked to HSD/EDS, especially in a younger population.

## Conclusions

This scoping review covering the last decade is the first review of its kind to provide a comprehensive summary all the reported symptoms and impact of EDS and HSD in an adult population. Gastroenterology, mood disorders and anxiety disorders were the most frequently reported physical and psychological manifestations whilst the social impact causes a disruption within their social networks and professional life. The current assessment and management of individuals with EDS/HSD is fragmented, with HCPs lacking sufficient knowledge of the condition. The findings of the review should be used to inform future work that promotes a multidisciplinary approach in clinical practice, improves the awareness and education of HCPs across relevant specialities, and encourages continuity of care.

## Data Availability

The data that support the findings of this review are available from the corresponding author upon reasonable request.

## Abbreviations

ADHD: Attention deficit hyperactivity disorder
AN: Anorexia nervosa
ANS: Autonomic nervous system
BJHS: Benign joint hypermobility syndrome
CWP: Chronic widespread pain
EDS: Ehlers-Danlos syndrome
ESS: Epworth Sleepiness Scale
GIQLI: Gastrointestinal Quality of Life Index
HADS: Hospital anxiety and depression scale
HCPs: Healthcare professionals
hEDS: Hypermobile Ehlers-Danlos syndrome
HSD: Hypermobility spectrum disorders
JHS: Joint hypermobility syndrome
MIDAS: Migraine disability assessment
MSK: Musculoskeletal
OSA: Obstructive sleep apnoea
PCC: Population, concept, context
PoTS: Postural tachycardia syndrome
P-QOL: Prolapse quality of life
PTSD: Post-traumatic stress disorder
QoL: Quality of life
SD: Standard deviation
TSK: Tampa Kinesiophobia Scale
UK: United Kingdom
USA: United States of America
